# Entero‐Articular Fistula Following Total Hip Arthroplasty in a Patient Post Pelvic Exenteration

**DOI:** 10.1155/cris/3978486

**Published:** 2026-07-30

**Authors:** Brendan Parnell, Raymond Hayler, Rayan Mourad, Ashish Vaska, David Morris, Robert Molnar

**Affiliations:** ^1^ Peritonectomy Unit, Department of Surgery, St George Hospital, Kogarah 2217, New South Wales, Australia, nsw.gov.au; ^2^ Faculty of Medicine and Health, The University of Sydney, Camperdown 2050, New South Wales, Australia, sydney.edu.au; ^3^ St George Hospital Clinical School, University of New South Wales, Kogarah 2217, New South Wales, Australia, unsw.edu.au; ^4^ Department of Orthopaedic Surgery, St George Hospital, Kogarah 2217, New South Wales, Australia, nsw.gov.au

**Keywords:** entero-articular fistula, pelvic exenteration, total hip arthroplasty

## Abstract

Entero‐articular fistulas are rare but serious complications following total hip arthroplasty (THA), often associated with prior abdominal or pelvic surgery, radiation therapy, or chronic steroid use. We report a case of a 65‐year‐old woman with metastatic ovarian cancer previously treated with pelvic exenteration and heated intra‐peritoneal chemotherapy (HIPEC), who developed an entero‐articular fistula following THA for avascular necrosis (AVN). Post‐operatively, she presented with systemic signs of infection and polymicrobial joint cultures containing enteric organisms, raising suspicion for bowel communication. Imaging revealed gas tracking from the pelvis to the hip joint, and subsequent surgery confirmed a fistulous connection with the small bowel adherent to the pelvic wall and protrusion of the allograft into the bowel. Management involved bowel resection, mesh placement, multiple joint washouts and prolonged antimicrobial therapy. This case highlights the diagnostic challenges of entero‐articular fistulas, particularly in patients with complex surgical histories and underscores the importance of a multidisciplinary approach for effective diagnosis and management.

## 1. Introduction

Entero‐articular fistulas are an abnormal connection between the bowel and a joint space. They are a rare complication following total hip arthroplasty (THA) with limited case reports in the literature. Abdominal surgery [1–3], history of radiation therapy [1, 4], and chronic steroid use [5] have been described as risk factors for fistula formation. Previous abdominal surgery predisposes patients to adhesion formation [6, 7], which may increase the risk of entero‐articular fistulation by close adherence of enteric structures to the pelvic sidewall [8]. This increases the risk of compromise during intra‐articular procedures if the joint wall is breached. These fistulas can lead to reduced mobility, pain, sepsis and THA revision [4].

This case report describes a 65‐year‐old female with a history of metastatic high‐grade serous ovarian cancer treated with pelvic exenteration and heated intra‐peritoneal chemotherapy (HIPEC). This patient subsequently developed avascular necrosis (AVN) of the right hip secondary to pelvic radiation therapy. This is an uncommon complication of pelvic radiation therapy, with incidence being reported as low as 0.5% in one paper [9]. With other papers reporting the incidence to be between 2.1% and 34% [10]. This patient subsequently underwent a right total hip replacement (THR), which was complicated post‐operatively by an entero‐articular fistula. This case reviews difficulties in diagnosis and emphasises the importance of a multidisciplinary approach to the management of prosthetic joint infections.

## 2. Case Presentation

A 65‐year‐old woman with a complex medical history presented for a right THR due to AVN. Her medical history included metastatic high‐grade serous ovarian cancer, diagnosed 7 years prior and treated with extensive surgery, including a total hysterectomy and bilateral salpingo‐oophorectomy, followed by radiation therapy. Three years post‐hysterectomy, the patient experienced cancer recurrence, and she underwent an anterior pelvic exenteration and HIPEC. A further 3 years post pelvic exenteration, the patient developed significant hip pain and impaired mobility. CT, MRI and bone scans were consistent with AVN of the right hip, likely secondary to radiation therapy. At the time of diagnosis of AVN, this patient was 3 years post her most recent pelvic exenteration with no evidence of active disease, suggesting a period of disease stability.

**Figure 1:**
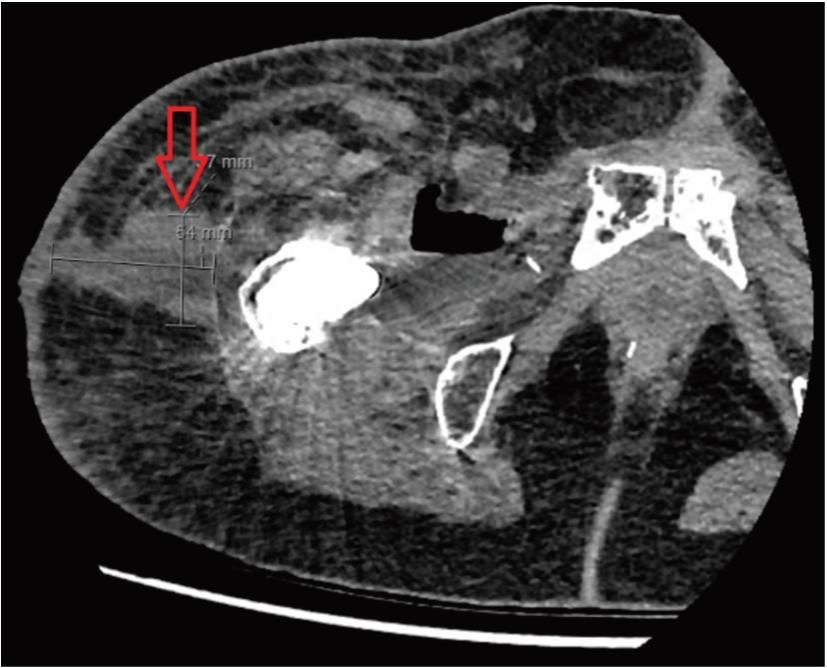
CT right hip, red arrow indicating fluid around the hip joint.

Given the patient’s deteriorating mobility and pain secondary to AVN, and in the context of her current disease remission, a joint decision was made by the orthopaedic and oncology teams to proceed with THA to optimise functional status and quality of life. This was performed via a posterior approach with a femoral head allograft to the defects in the acetabulum. The acetabulum was reamed, and a Zimmer G7 multi‐hole cup was inserted with 4 mm× 54 mm screws. A Zimmer MS‐30 femoral stem was then inserted into the femoral canal with a 36 mm −3.5 cobalt chrome femoral head. No complications were reported during this procedure. Five days post‐operatively, the patient developed a fever, tachycardia and hypotension (temperature 39°, HR 110 and systolic BP 88). Inflammatory markers demonstrated a CRP of 189. Initial CT scans of the chest, abdomen and pelvis were negative for a source of infection. Erythema, warmth, and tenderness around the surgical site raised concerns for a right prosthetic joint infection. An ultrasound showing fluid collection was aspirated, yielding 80 mL of purulent, malodorous fluid, which grew polymicrobial growth of *Streptococcus constellatus*, *Klebsiella variicola*, *Enterococcus faecium* and *Enterobacter cloacae*. Further CT imaging of the hip (Figure 1) confirmed fluid within the hip joint. The patient underwent a right hip washout, debridement, and head exchange, with cultures growing *E. faecium*, *Lacticaseibacillus rhamnosus*, *K. variicola* and *Candida albicans*. These are common pathogens found in the bowel and female genital tract, raising suspicion for fistulation. The patient was started on a regime of cefepime, micafungin and vancomycin in discussion with the Infectious disease team. A second right hip washout and debridement revealed a large volume of pus deep in the hip fascia, directly communicating with the hip prosthesis. Repeat cultures grew similar organisms to prior microscopy. CT hip and pelvis were performed with the suggestion of gas tracking from the anterior prosthetic hip joint into the pelvis, suspicious for a fistula. Frank faecal drainage from the patient’s right hip drain further indicated an entero‐articular fistula. A combined case of orthopaedic surgery and general surgery was performed. The patient underwent a laparotomy with intraoperative findings of extensive adhesions with the small bowel densely adherent to the vaginal wall and pelvic sidewall and the bone allograft protruding into the small bowel with purulent/faecal contamination. The entero‐articular fistula tract was excised, and the involved small bowel was resected, leaving 1.2 m of small bowel remaining past the duodenojejunal flexure. A biosynthetic absorbable mesh was secured to the pelvic sidewall to isolate the small bowel from the pelvis. This was followed by a right‐hip washout. Eight days post‐operatively, increasing erythema around the right hip wound site was identified. CT imaging of the hip identified a growing right hip collection, which prompted a further joint washout and debridement with the insertion of a new Zimmer 36 mm femoral head.

Due to the patient’s co‐morbidities and desire to minimise further major surgeries, Infectious disease input was sought to formulate a long‐term suppressive antibiotic regime for her chronically infected right hip in preference to a two‐stage revision approach. Choice of antibiotics was complicated due to antimicrobial resistance of cultured organisms, reduced absorption of oral antibiotics secondary to the amount of small bowel (1.2 m) and her polymicrobial growth. The patient was managed on clindamycin, meropenem, micafungin and vancomycin. Six weeks following her initial surgery, the patient was transitioned to fluconazole, trimethoprim/sulfamethoxazole and clindamycin. Fluconazole and trimethoprim/sulfamethoxazole were later discontinued, and clindamycin was continued for lifelong suppression. At follow‐up 6 months later, the patient’s wounds had healed well, bloods were remaining stable and her right hip was non‐irritable with a good range of motion.

## 3. Discussion

Entero‐articular fistulas are rare complications following THA, with limited case reports describing their occurrence. Past literature has described abdominal surgery [1–3], history of radiation therapy [1, 4] and chronic steroid use [5] as potential risk factors for fistula formation. The patient in this case had a history of significant abdominal surgeries, including cytoreductive surgery and HIPEC, resulting in increased abdominal adhesion formation [6, 7]. Along with her history of pelvic radiation, this may have predisposed her to having a higher risk of fistula formation following THA than other patients.

Tai et al. [11] reported 70% of prosthetic joint infections are monomicrobial, with the most common pathogens involved being *Staphylococcus aureus* and coagulase‐negative *Staphylococcus* species. When a sinus tract is present, the probability of a polymicrobial infection or gram‐negative bacteria being involved is greatly increased [11]. The organisms isolated in this case included *E. faecium*, *L. rhamnosus*, *K. variicola*, and *C. albicans*, all of which are commonly found in bowel and vaginal flora [12–14]. The presence of these atypical organisms in a polymicrobial pattern should prompt the consideration of a gastrointestinal or gynaecological source of infection, either by contamination or communication.

The early diagnosis of entero‐articular fistulas is an essential but challenging process since they can mimic other more common post‐operative complications such as infection or wound dehiscence and are rarely encountered. Drain monitoring for faecal or purulent output has been described as a method of detecting entero‐articular fistulas [4]. Further imaging techniques such as CT scans and sinograms may also assist in the detection of fistulas prior to surgical exploration [4, 15, 16]. In this case, the presence of faecal drainage from the patient’s hip joint provided evidence of an entero‐articular fistula.

A key learning point from this case is that entero‐articular fistulas in patients with complex oncological and surgical histories cannot be managed by a single speciality. The index procedure itself warranted pre‐operative multidisciplinary review to risk‐stratify the patient and anticipate post‐operative complications. The sequential involvement of orthopaedic surgery, general surgery, infectious diseases, and oncology teams in this case was central to achieving a favourable outcome. Surgeons performing arthroplasty in patients with prior pelvic or abdominal surgery should engage a multidisciplinary team early, both in the pre‐operative planning phase and at the first sign of atypical post‐operative infection.

A second learning point relates to the management of the resulting prosthetic joint infection. Standard practice for chronic prosthetic joint infection typically involves a two‐stage revision: removal of the implant, placement of an antibiotic spacer, a period of systemic antibiotic therapy and subsequent reimplantation once the infection is eradicated. However, in patients with significant co‐morbidities, prior pelvic surgery, short bowel syndrome and limited life expectancy, the morbidity of a two‐stage revision must be carefully weighed against the potential functional benefit. In this case, the multidisciplinary team elected for long‐term suppressive antibiotic therapy as a limb‐preserving, quality‐of‐life‐focused strategy. This case, therefore, highlights that individualised, patient‐centred decision‐making, informed by oncological status, functional goals and co‐morbidity burden, is essential when standard surgical algorithms are not appropriate.

## Funding

The authors received no financial support for the research, authorship and/or publication of this article. Open access publishing facilitated by The University of Sydney, as part of the Wiley ‐ The University of Sydney agreement via the Council of Australasian University Librarians.

## Ethics Statement

Our institution does not require ethical approval for reporting individual cases or case series.

## Consent

Written informed consent was obtained from the patient for the publication of this case report and any accompanying images, in accordance with the journal’s policy and CARE guidelines.

## Conflicts of Interest

The authors declare no conflicts of interest.

## Data Availability

The data that support the findings of this study are available from the corresponding author upon reasonable request.
